# Bioinformatic Analysis Reveals Genome Size Reduction and the Emergence of Tyrosine Phosphorylation Site in the Movement Protein of New World Bipartite Begomoviruses

**DOI:** 10.1371/journal.pone.0111957

**Published:** 2014-11-10

**Authors:** Eric S. Ho, Joan Kuchie, Siobain Duffy

**Affiliations:** 1 Department of Biology, Lafayette College, Easton, Pennsylvania, United States of America; 2 New Jersey City University, Jersey City, New Jersey, United States of America; 3 Department of Ecology, Evolution and Natural Resources, Rutgers University, New Brunswick, New Jersey, United States of America; Institute of Infectious Disease and Molecular Medicine, South Africa

## Abstract

Begomovirus (genus Begomovirus, family *Geminiviridae*) infection is devastating to a wide variety of agricultural crops including tomato, squash, and cassava. Thus, understanding the replication and adaptation of begomoviruses has important translational value in alleviating substantial economic loss, particularly in developing countries. The bipartite genome of begomoviruses prevalent in the New World and their counterparts in the Old World share a high degree of genome homology except for a partially overlapping reading frame encoding the pre-coat protein (PCP, or AV2). PCP contributes to the essential functions of intercellular movement and suppression of host RNA silencing, but it is only present in the Old World viruses. In this study, we analyzed a set of non-redundant bipartite begomovirus genomes originating from the Old World (N = 28) and the New World (N = 65). Our bioinformatic analysis suggests ∼120 nucleotides were deleted from PCP’s proximal promoter region that may have contributed to its loss in the New World viruses. Consequently, genomes of the New World viruses are smaller than the Old World counterparts, possibly compensating for the loss of the intercellular movement functions of PCP. Additionally, we detected substantial purifying selection on a portion of the New World DNA-B movement protein (MP, or BC1). Further analysis of the New World MP gene revealed the emergence of a putative tyrosine phosphorylation site, which likely explains the increased purifying selection in that region. These findings provide important information about the strategies adopted by bipartite begomoviruses in adapting to new environment and suggest future *in planta* experiments.

## Introduction

Begomoviruses (genus Begomovirus, family *Geminiviridae*) are single-stranded DNA viruses of dicots with small genomes - one or two circular segments of ∼2.5–2.9 K nucleotides (nts). Begomoviruses are transmitted by the whitefly *Bemisia tabaci* [1], [2] and their damaging infections pose a severe threat to commercial and subsistence production of key crops worldwide, including tomato, squash, cassava and bean [3]. Understanding the molecular biology and adaptation of begomoviruses to novel hosts has an important socioeconomic impact as they are emerging problems in developing countries [3]. The vast majority of begomovirus sequences also exhibit a classic biogeographic pattern: they fall into clades of New World (the Americas, and Caribbean) and Old World (rest of the world) viruses, with New World viruses thought to be derived from those in the Old World [4]–[6]. Bipartite begomoviruses, which have two similarly-sized, ambisense genomic segments termed DNA-A and DNA-B, are found worldwide, with monopartite begomoviruses largely restricted to the Old World [7]. The DNA-A segment contains five or six genes, including the capsid protein (CP, also known as AV1), the replication-associated protein (REP, also known as AC1), a transcriptional activator (TrAP, also known as AC2), a replication enhancer (REn, also known as AC3) that overlaps with both the REP and TrAP genes and a virulence factor (AC4) that overlaps the reading frame within REP. The DNA-B segment contains two non-overlapping genes: the nuclear shuttle protein (NSP, also known as BV1), and the movement protein (MP, also known as BC1).

Old and New World bipartite begomoviruses share a high degree of homology, with the largest exception being the gene for the pre-coat protein (PCP, also known as AV2), which partially overlaps the CP gene and is only present in Old World viruses [8]. PCP and the monopartite V2 has been shown to localize at the cell periphery and is thought to act as a “movement protein” by increasing the size exclusion limit of the plasmodesmata [9], [10]. They also suppresses RNA silencing by binding to the host’s SGS3 protein [11]. V2 is thought to be the key movement protein in monopartite Old World viruses, but two genes on the DNA-B segment (NSP and MP) also contribute to systemic infection of plants by bipartite begomoviruses [12]. Virulent New World begomoviruses must rely on their other seven proteins to cope with the loss of PCP, and this is frequently invoked as the reason the DNA-B segment is required for infectivity of the overwhelming majority of New World begomoviruses [13]. Despite this assumption, the selective pressures imposed by the loss of PCP on the remaining New World viral genes have not been examined.

In this report we have compared the genome size, degree of variability and purifying selection of the viral genes between the Old and New World. Results indicate a loss of 100 nts in PCP’s promoter region, stronger purifying selection on the two DNA-B genes in the New World, and the emergence of a putative tyrosine phosphorylation site in the New World MP. Studies with RNA plant viruses have shown that phosphorylation of MP regulates their localization and may account for cell-to-cell movement [14]. We speculate that the reduction in viral cell-to-cell movement caused by the loss of the PCP in the New World begomoviruses may be compensated by systematic genome size reduction and/or the gain of additional phosphorylation activity in the MP.

## Materials and Methods

### Compilation of bipartite begomovirus genomes

Genomes of begomovirus were downloaded from the June-2012 release of the viral genome database hosted in NCBI (ftp://ftp.ncbi.nih.gov/refseq/release/viral/). Only genomes containing the distinct, invariant nonamer “TAATATT|AC” were included in this study (the vertical bar represents the cleavage site). The pairing of DNA-A and DNA-B genomes, and the classification of genomes into Old and New Worlds were done semi-automatically according to the information stated in NCBI’s RefSeq records [15] and ICTV report [16]. To ease sequence comparison, the beginning of the cleavage site “AC” was adopted as reference position 1 and the original genomic coordinates stated in NCBI’s RefSeq records of the begomoviruses were adjusted accordingly. 33 and 83 Old and New World bipartite begomoviruses were collected, respectively, before further redundancy checking.

### Identification of common regions

The DNA-A and DNA-B genomes of a bipartite begomovirus share a 200- to 250-nt long highly identical segment (>85%), namely the common region (CR), in which the invariant nonamer “TAATATT|AC” resides near to the middle of it. To determine the 5′ and 3′ termini of the CR, a pair of segments consisting of 250 nts upstream and downstream flanking regions of the invariant nonamer from DNA-A and DNA-B was aligned. Based on the alignment, the longest stretch of highly identical (at least 20 nts long and 80% identity) segment flanking the invariant nonamer was taken as the CR.

### Identification of non-redundant genomes and ORFs

We clustered DNA-As together if their CRs shared >80% similarity. If more than one species was found in a cluster, only one species was retained arbitrarily for further analysis. A Peruvian begomovirus, Tomato leaf deformation virus (ToLDeV), was confirmed to be the first New World monopartite begomovirus in 2013 [7], but this was after our dataset had been finalized. ToLDeV does not appear to have a PCP gene. As a result, 26 out of 33 (85%) and 65 out of 83 (78%) non-redundant Old World and New World bipartite begomovirus genomes were included in this analysis (Table 1). The full list of bipartite begomovirus genomes used and their sizes can be found in the Table S1 in File S1. Additionally, ORFs specified in RefSeq records were verified. We required the stated coding sequences or ORFs be translated exactly to the protein sequences specified in the RefSeq records. Genes failed to meet this requirement were excluded from this study (Table 1). Genomes and viral protein sequences used in this study can be downloaded as Data S1 or through this web link: http://sites.lafayette.edu/hoe/files/2014/01/bipartite_seqs_eh_jk_sd.tar_.gz.

**Table 1:** Number of bipartite begomovirus genomes and proteins included in this study.

	Old World (28)	New World (65)
Coat protein (CP/AV1)	28	65
Pre-coat protein (PCP/AV2)	23	0
Replication-associated protein (REP/AC1)	28	65
Transcription activator protein (TrAP/AC2)	27	63
Replication enhancer (REn/AC3)	27	64
AC4	26	42
Nuclear shuttle protein (NSP/BC1)	28	65
Movement protein (MP/BV1)	28	64

The bracketed numbers in the column head is the number of genomes included in this report. Note that not all genes from included genomes were automatically accepted in this study because we found some of the ORFs documented in NCBI RefSeq database showed discrepancies with the associated protein sequences.

### dN/dS calculation

dN/dS represents the log ratio of the rate of non-synonymous substitutions to the rate of synonymous (silent) substitutions. A negative, zero, or positive dN/dS value indicates purifying (negative), neutral, or positive selection, respectively. Protein sequences were aligned by T_COFFEE [17] using default parameters. Protein alignments were converted to codon alignments using pal2nal v14 [18]. The codon alignments were submitted to the tool SLAC [19] hosted in the Datamonkey web server http://www.datamonkey.org/ [20] for site dN/dS calculation. Substitution models were selected by iterating the likelihood ratio tests between nested and non-nested models. This procedure is implemented in Datamonkey web server and detailed discussion of the procedure can be found in [21]. The results calculated by SLAC were downloaded in CSV format for analysis.

### Pairwise protein sequence alignment

As dynamic programming approach to local pairwise sequence alignment produces the optimal alignment for a given scoring scheme. We used the percentage of identity calculated by an implementation of such approach i.e. Smith-Waterman water program [22], to determine the diversity of each viral protein for either Old or New World regions. BLOSUM62 score matrix was used and gap opening penalty and gap extension penalty were 10 and 0.5, respectively.

### D-statistic of the Kolmogorov-Smirnov test

In order to ascertain the statistical significance of the difference between two non-Gaussian, cumulative distributions of protein sequence similarities and dN/dS values, we quantified the difference using the D-statistic of the two-sample Kolmogorov-Smirnov (KS) test. In both worlds, the viral protein AC4 exhibited the highest diversity. Thus, AC4 was chosen as the reference for two-sample D-statistic calculation. D-statistics were computed using the R function ks. test () [23]. All the values of D-statistic calculated showed significant differences between the two worlds with p-value in the range of 10^−16^.

### Scanning of functional sites in the movement protein

We developed a Python script (available upon request) to scan for functional sites in protein sequences using the BioPython scanProsite package [24], where the option for skipping of high probability of occurrence was turned off. In addition, our script used the bootstrap approach to compute the p-value of hits through these steps: 1. Obtain the list of functional sites detected in the input sequences through scanProsite, 2. Scramble input sequences, 3. Scan for functional sites in scrambled sequences, 4. Register the list of functional sites found in scrambled sequences, 5. Repeat steps 2 to 4 100 times (a user-defined parameter), 6. Estimate the p-value of a functional site by dividing the occurrence of the functional site in scrambled sequences by the occurrence of the same site in the original input sequences.

## Results and Discussion

### New World begomoviruses have smaller segments

**Figure 1:**
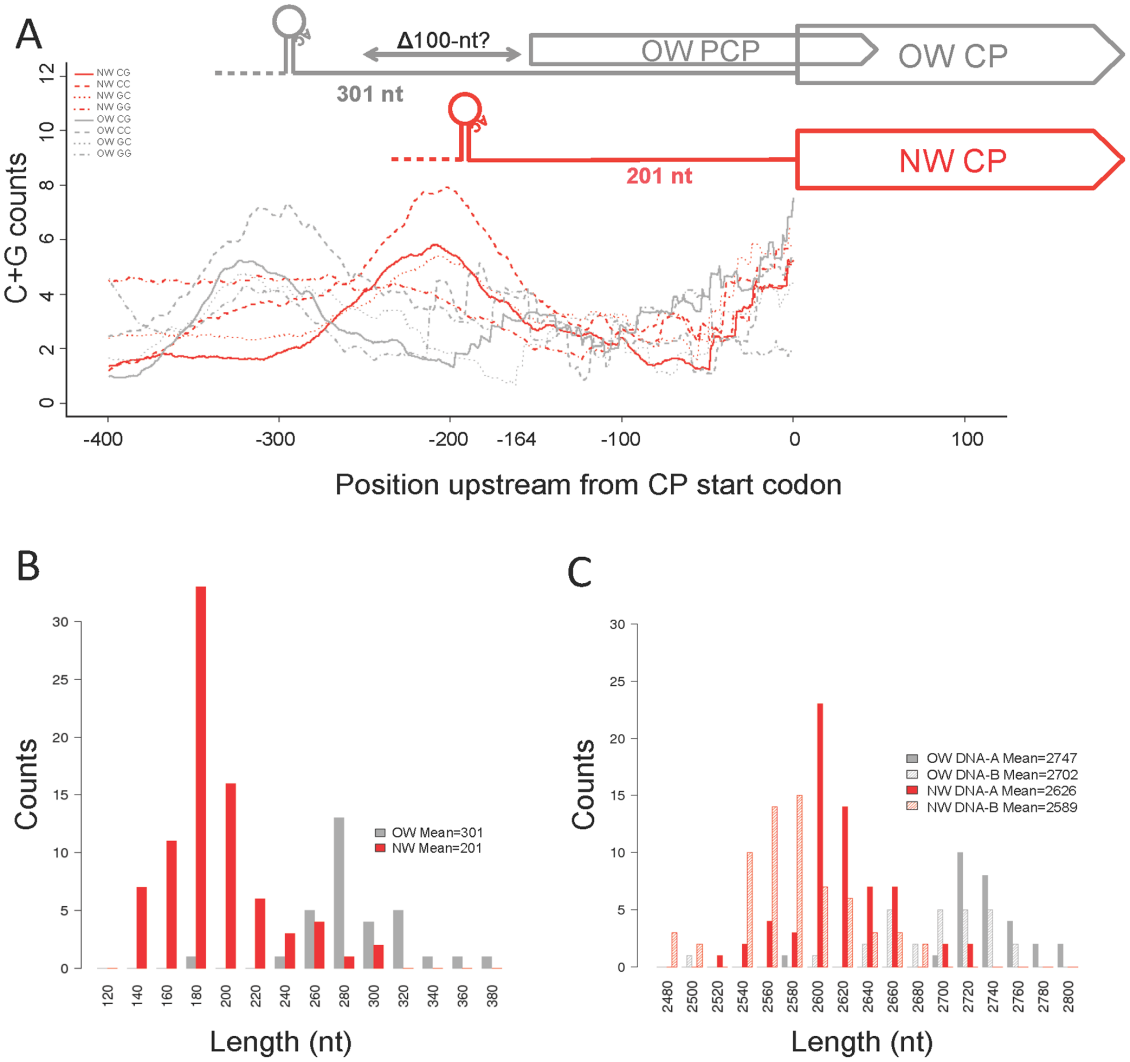
The loss of 100 nts from the promoter region of the New World PCP. A) C+G profiles of the homologous regions upstream from the CP from Old and New World bipartite begomoviruses. All positions labeled in the gene structure diagram are average values. Each plot represents the average number of CC, CG, GC, or GG in a 60-nt window. B) Distributions of the distance between the cleavage site “AC” of the invariant nonamer and the beginning of CP gene. C) Distribution of genome size.

**Figure 2:**
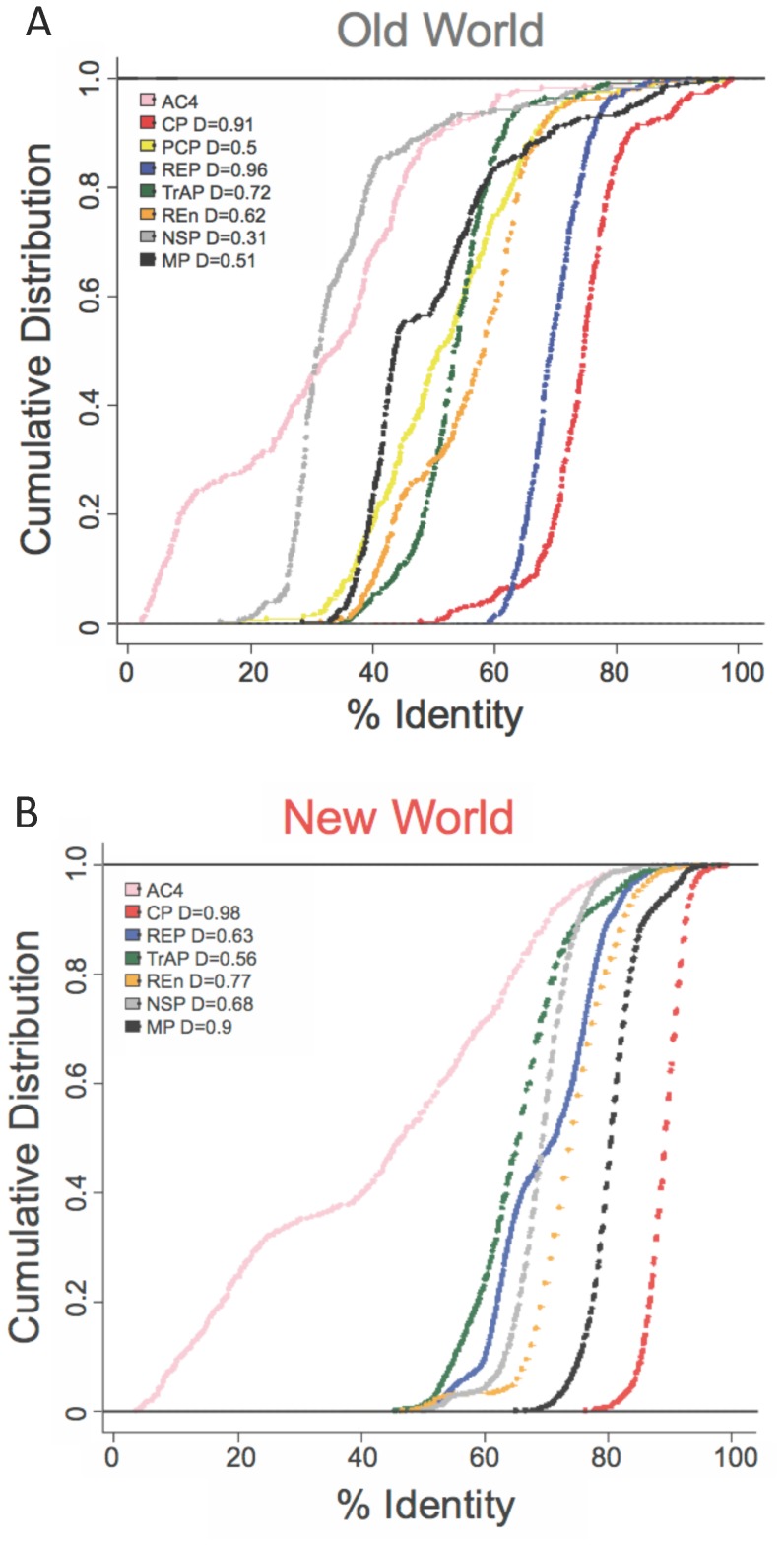
Protein sequences variability by gene. A) Cumulative distributions of percentage of identity (%id) of viral proteins from the Old World bipartite begomoviruses. The D values printed beside the protein name in the legend represent the magnitude of deviation of the plot from the AC4’s curve and it was determined by two-sample Kolmogorov test. Larger the D value, the great is the deviation from AC4. B) Proteins from the New World.

We discovered that the genome size of DNA-A in the New World is on average 121 nts shorter than their counterparts in the Old World (Figure 1C). Intriguingly, though no apparent gene loss event was reported previously in the New World DNA-B, their genomes (mean size is 2,589 nts, standard deviation or s.d. 43) are also on average 113 nts smaller than the Old World DNA-B (mean is 2,702 nts and s.d. 55) as shown in Figure 1C. This commensurate genome size reduction does not seem to be coincidental as bipartite genome segments (DNA-A and DNA-B) in the New World begomoviruses show a higher correlation in size (R = 0.91, p-value <2.2×10^−16^) than those in the Old World (R = 0.74, p-value <8.1×10^−6^). Besides, the genome size differences between DNA-A and DNA-B concurred this point as we found smaller and less variable differences between DNA-A and DNA-B in the New World (mean 37 nts, s.d. 18) than the Old World (mean 45 nts, s.d. 37). Regardless of the geographical factor, this result may suggest size codependency of the bipartite genomes, which is still largely unknown. Our findings are unlikely confounded by biased samples as viral genes AC4 (pink) and REP (blue) exhibit similar spectra of sequence diversity between the two worlds (Figure 2). We further investigated whether or not deletions are localized at a particular region and how it may explain the loss of PCP in the New World begomoviruses.

### Deletions are localized at PCP’s promoter region

We compared dinucleotide profiles in the 400-nt upstream, homologous region of all viral genes using a 60-nt sliding window between the two worlds (see Materials and Methods, and Figure S1A–G in File S1). We found that dinucleotides were better than single nucleotides in insulating the profiles from random nucleotide fluctuation. If short (<5 nts) insertions or deletions are scattered, dinucleotide profiles between the two worlds should exhibit similar patterns; otherwise we should see a direct shift between the two profiles. Among dinucleotide profiles of all genes, only C+G profiles, i.e. CC, CG, GC, and CG, of the CP gene were found to differ between the two worlds in which the region with high concentration of C+G in the New World was shifted ∼100 nts closer to the start of the ORF (Figure 1A). The elevated C+G content is chiefly due to the stem of the highly conserved hairpin structure found in all begomoviruses in which the loop region contains the invariant “TAATATT|AC” nonamer (“|” represents the cleavage site during complementary strand synthesis). Corroborating results were found when we examined the distance between the cleavage site “AC” and the start of the CP genes in both worlds (Figure 1B) where the New World’s CP gene is on average 100 nts closer to the cleavage site “AC” than those in the Old World. This accounts for much of the 121 nts shorter average genome size of DNA-A of the New World viruses compared to those of the Old World (Figure 1C). In New World begomoviruses the distance from the cleavage site to CP is highly correlated with DNA-A size (R = 0.93), but to a lesser extent in the Old World (R = 0.72). Additionally, the C+G content in the non-overlapping region of PCP (from −164 to 0 in Figure 1A) remains at similar level between the two worlds. Therefore our analysis indicates one or more deletions totaling more than 100 nts were mainly localized in the proximal promoter region of PCP, not in other genomic regions, and that these deletions may have led to PCP inactivation in the New World begomoviruses.

Currently, little is known about the effect of genome size on cell-to-cell transport through plasmodesma but studies have shown that the plasmodesmata impose a size limit [25], [26]. Effective shuttling of viral genomes between cells without passing through the cell wall is critical for maintaining infectivity of plant viruses as small viruses do not encode enzymes to breakdown the cell wall, which other phytopathogens such as fungi employ [27]. This finding suggests genome size reduction may be one of the evolutionary paths selected for in the New World begomoviruses in order to maintain virulence despite the loss of cell-to-cell movement conferred by PCP.

### New World NSP and MP are under enhanced purifying selection

The compact begomovirus genome encodes only a small number of highly overlapping genes in ambisense, most known to have multiple functions during infection. The lost functions of the PCP gene are likely compensated by remaining genes in New World begomoviruses. Therefore, we took a comparative approach to identify the presence of purifying selection in the New World viral proteins. We measured the within-world diversity of each gene by pairwise protein sequence alignment. A high sequence similarity indicates strong conservation pressures on the genes. Figure 2 shows the cumulative distributions of pairwise identity (%id) of seven or eight viral proteins from the two worlds. In the Old World (Figure 2A), AC4 exhibits the highest variability followed by the two DNA-B proteins NSP and MP, then REn, TrAP, REP and finally CP. The CP is known to be the most conserved of all begomovirus proteins, and under the greatest amount of purifying selection [12], [28]. All distributions were tested for statistical significance (p-value ∼10^−26^) according to the two-sample Kolmogorov-Smirnov test with AC4 as the reference protein. Results from the New World viral proteins show much lower levels of diversity except for AC4 and REP. Such results are consistent with the presumed more recent origin of New World begomoviruses [4], but show a different pattern in protein variability (Figure 2B). The seven proteins are still bounded by AC4 as the most variable protein, and CP being the most conserved. The most striking difference is in the reduced variability of the New World MP (black plot in Figure 2B), which has become the most conserved protein after CP. Additionally, the New World NSP (gray plot in Figure 2B) is also found to show significant reduction in variability, comparable to the essential replication protein REP (blue plot in Figure 2B). It appears that both DNA-B genes are under stronger selective pressure in the absence of PCP. These results suggest that the less genomically compressed DNA-B genomic segment was more able to accommodate new or enhanced functions than the more constrained DNA-A segment, which already has several overlapping open reading frames.

**Figure 3:**
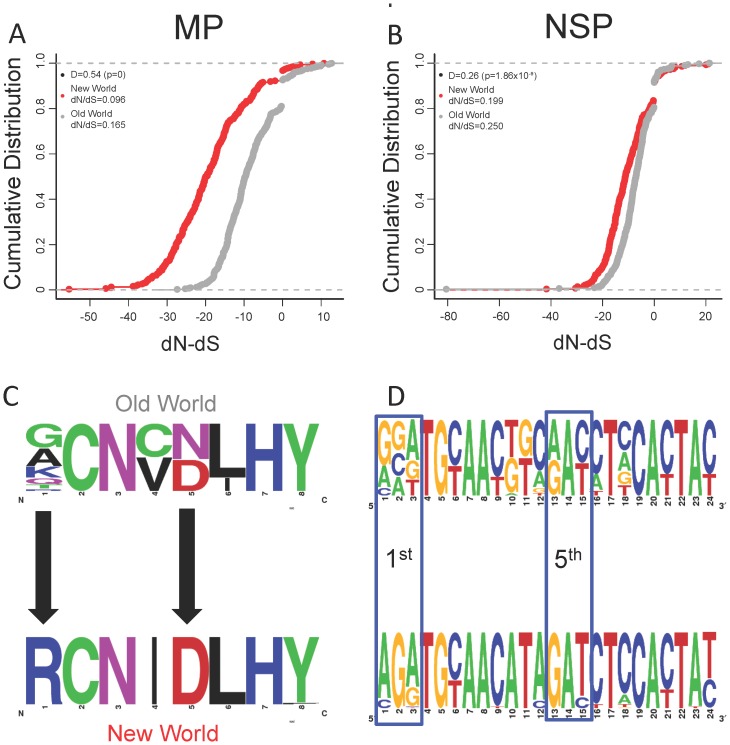
Cumulative distributions of site dN/dS values of the viral proteins. D-value, with p-value, represents the deviation of the New World curve from the Old World curve. D-value was calculated by the two-sample Kolmogorov test. Averaged site dN/dS is displayed on the top left, which reflects the overall selection pressure on the protein. A) MP. B) NSP. C) The emergence of putative tyrosine phosphorylation site in the New World MP. Consensus sequence pictures were created using Weblogo [34]. Searching is based on PROSITE database [35]. PROSITE ID of the tyrosine phosphorylation site is PS00007 where its consensus is [RK]-x(2,3)-[DE]-x(2,3)-Y. D) Codon alignment of the homologous region of the site.

To further confirm this point, we sought evidence for adaptive evolution at the nucleotide level to corroborate these protein sequence analyses. Adaptive evolution is measured by the log ratio of the rate of non-synonymous substitutions versus synonymous substitutions (dN/dS). If the rate of non-synonymous substitution is lower than synonymous substitution, dN/dS will yield a negative value, indicating amino acid substitution is unfavorable. Conversely, a positive dN/dS value indicates amino acid substitution is permissible, suggesting the protein is under positive or adaptive selection. Aligned protein sequences were converted to corresponding codon alignments before dN/dS calculation using the Single Likelihood Ancestor Counting (SLAC) method from the Datamonkey website [20], [29]. Figure 3A–B show the cumulative dN/dS ratios for MP and NSP. The New World MP shows the biggest deviation (D = 0.54, p-value = 0) from the Old World counterpart and nearly all dN/dS values fall in the negative region, reconfirming elevated purifying selection in the New World MP. But we did not see this in other viral genes (Figure S2 in File S1). We further explored whether or not purified residues in the New World MP constitute to any functional motif(s).

### The emergence of tyrosine phosphorylation site in the New World MP

In order to uncover the specific nucleotides subjected to elevated purifying selection in the New World MP, we compared the functional sites of MP in both worlds. Among all functional sites discovered, a putative tyrosine phosphorylation site [RK]-x(2,3)-[DE]-x(2,3)-Y (PROSITE ID: PS00007; the notation means the site starts with either R or K, followed by any 2 to 3 residues, and then a D or E residue, followed by any 2 to 3 residues, and ends with Y) shows the greatest difference between the two worlds: 62/65 New World MP sequences were found to carry the tyrosine phosphorylation site compared to only 1/28 from the Old World.

We also evaluated the likelihood for the emergence of this site by comparing the putative tyrosine phosphorylation site in the New World MP with the homologous region in the Old World MP. We aligned the eight-residue site and the corresponding codons. The amino acid consensus of the Old World (Figure 3C) shows only two residue substitutions are needed to transform the functionally indeterminate eight-residue site in the Old World MP to the tyrosine phosphorylation site found in the New World MP. From the codon perspective, four nucleotide substitutions from the first and fifth codons are sufficient to transform the site (Figure 3D).

## Conclusions

Our analysis strongly suggests one or more deletions of 100 nts in the promoter region of the New World PCP, which may be linked to the inactivation of PCP. The resultant shrunken genome may have had an advantage in cell-to-cell movement through plasmodesmata. Furthermore, our genome size analysis unraveled putative size codependency of the bipartite genomes. As the New World begomoviruses are presumably originated from the Old World counterparts recently [4], the conspicuous correlation between the New World DNA-A and DNA-B could be alluded to bottleneck effect. However, the more diverse Old World begomoviruses still maintain a high level of correlation (R = 0.74, p-value <8.1×10^−6^) between segment size of their bipartite genomes. DNA-B’s functional sequences – the common region (∼200 nts), ORFs of NSP (∼800 nts) and MP (∼900 nts) and their promoter regions (∼200 nts) – occupy ∼2,100 nts of the 2,700-nt genome on average, leaving ∼600 nts (22% of the genome) available for size reduction. According to our data (Table S1 in File S1), the mean, median and maximum difference between the bipartite genomes of the Old World viruses are only 45, 40, and 170 nts, respectively, which are far smaller than the 600 nts permissible range without interrupting the genomic structure of the viruses. This result is surprising as begomoviruses are fast mutating [30] and recombining [31], [32] ssDNA viruses, indicating the presence of unknown constraints that limit the variance in size between segments in bipartite genomes.

Our prediction aligns with findings in closely related monopartite begomoviruses. PCP has been reported previously to perform some MP functions, such as intracellular movement and cell periphery localization [9], [25], [26]. Additionally, in-vitro phosphorylation activity was reported in MP of Abutilon mosaic virus [33]. Our thorough bioinformatic comparison of geographically separated begomovirus species has produced a candidate region for detailed wet lab analysis. If the tyrosine phosphorylation site is critical to infectivity of New World begomoviruses, it will be a novel target for sequence-specific, anti-viral strategies.

## Supporting Information

File S1 Contains the following files: **Table S1:** Selected bipartite begomoviruses and their genome size. **Figure S1:** Dinucleotide profiles in 400-nt upstream regions. Window size is 60 nts. Y-axis denotes the average occurrences of the specified dinucleotide in the 60-nt window. Plots of dinucleotides AA, AC, …, TG, TT are arranged from top left to bottom right. **Figure S2:** Cumulative dN/dS values by gene.
(DOC)

Data S1 Genomes and protein sequences used in this study.
(TAR.GZ)
