# Supplementary material for: Bioinformatic Analysis Reveals Genome Size Reduction and the Emergence of Tyrosine Phosphorylation Site in the Movement Protein of New World Bipartite Begomoviruses
Source: PLoS One. 2014 Nov 10;9(11):e111957. doi: 10.1371/journal.pone.0111957 (PMC4226511; doi:10.1371/journal.pone.0111957)
Supplement: File S1 — Contains the following files: Table S1: Selected bipartite begomoviruses and their genome size. Figure S1: Dinucleotide profiles in 400-nt upstream regions. Window size is 60 nts. Y-axis denotes the average occurrences of the specified dinucleotide in the 60-nt window. Plots of dinucleotides AA, AC, …, TG, TT are arranged from top left to bottom right. Figure S2: Cumulative dN/dS values by gene. (DOC) [file pone.0111957.s001.doc]

**Supporting Information**

**Bioinformatic Analysis Reveals Genome Size Reduction And The Emergence Of Tyrosine Phosphorylation Site In The Movement Protein Of New World Bipartite Begomoviruses**

Eric S. Ho^1,*^, Joan Kuchie^2^, Siobain Duffy^3^

^1^ Department of Biology, Lafayette College, Easton, Pennsylvania, United States of America

^2^ New Jersey City University, Jersey City, New Jersey, United States of America

^3^ Department of Ecology, Evolution and Natural Resources, Rutgers University-New Brunswick, New Jersey, United States of America

***Corresponding author:** [hoe@lafayette.edu](mailto:hoe@lafayette.edu) (ESH)

**Table S1:** **Selected bipartite begomoviruses and their genome size**

| | **World** | **Name** | **DNA-A** | **A_size** | **DNA-B** | **B_size** | **A_size-B_size** | | --- | --- | --- | --- | --- | --- | --- | | NW | Abutilon Brazil virus DNA A, complete genome. | NC_014138 | 2653 | NC_014139 | 2618 | 35 | | NW | Abutilon mosaic Bolivia virus DNA-A, complete genome. | NC_015045 | 2717 | NC_015048 | 2682 | 35 | | NW | Abutilon mosaic Brazil virus DNA-A, complete genome. | NC_016574 | 2649 | NC_016577 | 2633 | 16 | | NW | Abutilon mosaic virus DNA A, complete sequence. | NC_001928 | 2632 | NC_001929 | 2585 | 47 | | NW | Bean calico mosaic virus DNA A, complete sequence. | NC_003504 | 2603 | NC_003505 | 2572 | 31 | | NW | Bean chlorosis virus isolate Venezuela:La Barinesa 459:2006 segment DNA-A | NC_019569 | 2647 | NC_019568 | 2632 | 15 | | NW | Bean dwarf mosaic virus DNA A, complete genome. | NC_001931 | 2615 | NC_001930 | 2576 | 39 | | NW | Bean golden mosaic virus DNA A, complete sequence. | NC_004042 | 2617 | NC_004043 | 2580 | 37 | | NW | Bean golden yellow mosaic virus DNA A, complete sequence. | NC_001439 | 2647 | NC_001438 | 2585 | 62 | | NW | Blainvillea yellow spot virus DNA-A, complete genome. | NC_010837 | 2666 | NC_010838 | 2634 | 32 | | NW | Blechum interveinal chlorosis virus DNA A, complete genome. | NC_019035 | 2645 | NC_019036 | 2640 | 5 | | NW | Cabbage leaf curl virus DNA A, complete sequence. | NC_003866 | 2583 | NC_003887 | 2513 | 70 | | NW | Chino del tomate virus DNA A, complete sequence. | NC_003830 | 2634 | NC_003831 | 2579 | 55 | | NW | Cleome leaf crumple virus DNA-A, complete genome. | NC_016578 | 2725 | NC_016572 | 2661 | 64 | | NW | Corchorus yellow spot virus DNA A, complete sequence. | NC_008492 | 2606 | NC_008493 | 2589 | 17 | | NW | Corchorus yellow vein virus - [Hoa Binh] DNA A, complete genome. | NC_006358 | 2724 | NC_006359 | 2691 | 33 | | NW | Cotton leaf crumple virus DNA A, complete sequence. | NC_004580 | 2630 | NC_004581 | 2550 | 80 | | NW | Dalechampia chlorotic mosaic virus isolate Venezuela:Albarico 1020:2007 segment DNA-A | NC_018716 | 2616 | NC_018718 | 2598 | 18 | | NW | Desmodium leaf distortion virus DNA A, complete sequence. | NC_008494 | 2569 | NC_008495 | 2514 | 55 | | NW | Dicliptera yellow mottle virus DNA A, complete sequence. | NC_003856 | 2607 | NC_003857 | 2597 | 10 | | NW | Euphorbia mosaic virus-Yucatan Peninsula DNA A, complete sequence. | NC_008304 | 2613 | NC_008305 | 2602 | 11 | | NW | Euphorbia yellow mosaic virus DNA A, complete genome. | NC_012553 | 2609 | NC_012554 | 2578 | 31 | | NW | Macroptilium mosaic Puerto Rico virus DNA A, complete sequence. | NC_004097 | 2615 | NC_004098 | 2571 | 44 | | NW | Macroptilium yellow mosaic Florida virus DNA A, complete sequence. | NC_004099 | 2642 | NC_004100 | 2605 | 37 | | NW | Macroptilium yellow mosaic virus DNA A, complete sequence. | NC_010647 | 2630 | NC_010648 | 2593 | 37 | | NW | Macroptilium yellow net virus DNA A, complete genome. | NC_017001 | 2620 | NC_017002 | 2577 | 43 | | NW | Melon chlorotic mosaic virus DNA-A, complete genome. | NC_014380 | 2628 | NC_014381 | 2607 | 21 | | NW | Merremia mosaic Puerto Rico virus DNA A, complete genome. | NC_015490 | 2640 | NC_015491 | 2585 | 55 | | NW | Merremia mosaic virus DNA A, complete sequence. | NC_007965 | 2557 | NC_007966 | 2492 | 65 | | NW | Okra mottle virus - [Brazil:okra] DNA A, complete sequence. | NC_011181 | 2660 | NC_011182 | 2653 | 7 | | NW | Okra yellow mosaic Mexico virus DNA A, complete genome. | NC_014066 | 2612 | NC_014067 | 2582 | 30 | | NW | Passionfruit severe leaf distortion virus DNA-A, complete genome. | NC_012786 | 2672 | NC_012787 | 2644 | 28 | | NW | Pepper golden mosaic virus DNA A, complete sequence. | NC_004101 | 2613 | NC_004096 | 2595 | 18 | | NW | Pepper huasteco yellow vein virus DNA A, complete sequence. | NC_001359 | 2631 | NC_001369 | 2589 | 42 | | NW | Potato yellow mosaic virus DNA A, complete sequence. | NC_001934 | 2593 | NC_001935 | 2547 | 46 | | NW | Rhynchosai mild mosaic virus DNA-A, complete genome. | NC_015488 | 2611 | NC_015489 | 2551 | 60 | | NW | Rhynchosia golden mosaic virus DNA A, complete sequence. | NC_010294 | 2604 | NC_010293 | 2551 | 53 | | NW | Sida golden mosaic Costa Rica virus DNA A, complete sequence. | NC_004657 | 2605 | NC_004658 | 2587 | 18 | | NW | Sida golden mosaic Florida virus-Malvastrum DNA-A, complete genome. | NC_014446 | 2608 | NC_014447 | 2578 | 30 | | NW | Sida golden mosaic Honduras virus DNA A, complete sequence. | NC_004659 | 2603 | NC_004660 | 2589 | 14 | | NW | Sida golden yellow vein virus-[Jamaica:Liguanea2:2008] DNA-A, complete genome. | NC_004635 | 2608 | NC_014799 | 2542 | 66 | | NW | Sida micrantha mosaic virus segment A, complete sequence. | NC_005330 | 2675 | NC_005331 | 2656 | 19 | | NW | Sida mosaic Alagoas virus DNA-A, complete genome. | NC_016573 | 2678 | NC_016579 | 2614 | 64 | | NW | Sida mosaic Bolivia virus 1 DNA-A, complete genome. | NC_015046 | 2680 | NC_015044 | 2668 | 12 | | NW | Sida mosaic Bolivia virus 2 DNA-A, complete genome. | NC_015043 | 2679 | NC_015047 | 2637 | 42 | | NW | Sida yellow mosaic Yucatan virus DNA A, complete sequence. | NC_008779 | 2611 | NC_008780 | 2586 | 25 | | NW | Sida yellow mottle virus DNA-A, complete genome. | NC_016082 | 2621 | NC_016083 | 2601 | 20 | | NW | Soybean chlorotic spot virus DNA-A, complete sequence. | NC_018457 | 2623 | NC_018456 | 2585 | 38 | | NW | Squash leaf curl virus A component DNA, complete sequence. | NC_001936 | 2634 | NC_001937 | 2607 | 27 | | NW | Squash mild leaf curl virus-[Imperial Valley] DNA A, complete sequence. | NC_004645 | 2612 | NC_004646 | 2578 | 34 | | NW | Tobacco yellow crinkle virus DNA A, complete genome. | NC_015627 | 2601 | NC_015628 | 2553 | 48 | | NW | Tomato chlorotic mottle virus DNA A, complete sequence. | NC_003664 | 2622 | NC_003665 | 2573 | 49 | | NW | Tomato common mosaic virus DNA-A, complete genome. | NC_010835 | 2560 | NC_010836 | 2498 | 62 | | NW | Tomato dwarf leaf virus DNA-A, complete genome. | NC_016580 | 2540 | NC_016581 | 2494 | 46 | | NW | Tomato golden mottle virus DNA A, complete sequence. | NC_008058 | 2614 | NC_008057 | 2558 | 56 | | NW | Tomato mild mosaic virus DNA-A, complete genome. | NC_010833 | 2708 | NC_010834 | 2663 | 45 | | NW | Tomato mild yellow leaf curl Aragua virus DNA A, complete sequence. | NC_009490 | 2609 | NC_009491 | 2559 | 50 | | NW | Tomato mottle Taino virus DNA A, complete sequence. | NC_001828 | 2597 | NC_001917 | 2562 | 35 | | NW | Tomato rugose mosaic virus DNA A, complete sequence. | NC_002555 | 2622 | NC_002556 | 2572 | 50 | | NW | Tomato yellow leaf distortion virus DNA A, complete genome. | NC_011348 | 2630 | NC_017913 | 2589 | 41 | | NW | Tomato yellow margin leaf curl virus DNA A, complete genome. | NC_005852 | 2575 | NC_005853 | 2543 | 32 | | NW | Tomato yellow mottle virus segment DNA-A, complete sequence. | NC_019946 | 2574 | NC_019947 | 2547 | 27 | | NW | Tomato yellow spot virus DNA-A, complete genome. | NC_007726 | 2674 | NC_007727 | 2626 | 48 | | NW | Tomato yellow vein streak virus DNA-A, complete genome. | NC_010949 | 2561 | NC_010950 | 2569 | -8 | | NW | Wissadula golden mosaic St Thomas Virus DNA A, complete sequence. | NC_010948 | 2622 | NC_010951 | 2578 | 44 | | OW | African cassava mosaic virus DNA A, complete sequence. | NC_001467 | 2779 | NC_001468 | 2724 | 55 | | OW | Asystasia begomovirus 1 DNA-A, complete genome. | NC_016575 | 2727 | NC_016576 | 2661 | 66 | | OW | Cassava mosaic Madagascar virus DNA A, complete genome. | NC_017004 | 2769 | NC_017005 | 2741 | 28 | | OW | Clerodendrum golden mosaic China virus DNA A, complete genome. | NC_011346 | 2776 | NC_011347 | 2739 | 37 | | OW | Clerodendrum golden mosaic virus DNA-A, complete sequence. | NC_010713 | 2767 | NC_010714 | 2757 | 10 | | OW | East African cassava mosaic Cameroon virus DNA A, complete sequence. | NC_004625 | 2802 | NC_004630 | 2741 | 61 | | OW | East African cassava mosaic Kenya virus DNA A, complete genome. | NC_011583 | 2797 | NC_011584 | 2776 | 21 | | OW | East African cassava mosaic Zanzibar virus DNA-A, complete genome. | NC_004655 | 2785 | NC_004656 | 2763 | 22 | | OW | Gossypium punctatum mild leaf curl virus DNA A, complete genome. | NC_011805 | 2737 | NC_012120 | 2725 | 12 | | OW | Horsegram yellow mosaic virus, complete genome. | NC_005635 | 2728 | NC_005636 | 2677 | 51 | | OW | Indian cassava mosaic virus DNA A, complete sequence. | NC_001932 | 2815 | NC_001933 | 2645 | 170 | | OW | Kudzu mosaic virus DNA-A, complete sequence. | NC_009645 | 2731 | NC_009647 | 2672 | 59 | | OW | Loofa yellow mosaic virus DNA A, complete sequence. | NC_004824 | 2742 | NC_004825 | 2713 | 29 | | OW | Mungbean yellow mosaic India virus DNA A, complete sequence. | NC_004608 | 2745 | NC_004609 | 2616 | 129 | | OW | Mungbean yellow mosaic virus DNA A, complete sequence. | NC_001983 | 2723 | NC_001984 | 2675 | 48 | | OW | Pepper yellow leaf curl Indonesia virus DNA-A, complete genome. | NC_008283 | 2750 | NC_008284 | 2726 | 24 | | OW | Ramie mosaic virus DNA-A, complete genome. | NC_010791 | 2738 | NC_010792 | 2708 | 30 | | OW | Rhynchosia yellow mosaic India virus DNA-A, complete genome. | NC_014902 | 2727 | NC_014903 | 2679 | 48 | | OW | Soybean chlorotic blotch virus DNA A, complete genome. | NC_014141 | 2708 | NC_014142 | 2647 | 61 | | OW | Squash leaf curl China virus - [B] DNA-A, complete genome. | NC_007339 | 2736 | NC_007338 | 2719 | 17 | | OW | Squash leaf curl Philippines virus segment A, complete genome. | NC_005845 | 2739 | NC_005846 | 2705 | 34 | | OW | Sri Lankan cassava mosaic virus DNA A, complete sequence. | NC_003861 | 2755 | NC_003862 | 2711 | 44 | | OW | Tomato golden mosaic virus DNA A, complete sequence. | NC_001507 | 2588 | NC_001508 | 2508 | 80 | | OW | Tomato leaf curl Gujarat virus - [Varanasi] segment A, complete sequence. | NC_004558 | 2757 | NC_004559 | 2688 | 69 | | OW | Tomato leaf curl New Delhi virus DNA A, complete sequence. | NC_004611 | 2739 | NC_004612 | 2696 | 43 | | OW | Tomato yellow leaf curl Kanchanaburi virus DNA A, complete sequence. | NC_005812 | 2752 | NC_005811 | 2752 | 0 | | OW | Tomato yellow leaf curl Thailand virus DNA A, complete sequence. | NC_000869 | 2751 | NC_000870 | 2737 | 14 | | OW | Watermelon chlorotic stunt virus DNA A, complete sequence. | NC_003708 | 2745 | NC_003709 | 2753 | -8 | |  |  |  |  |  |
| --- | --- | --- | --- | --- | --- | --- | --- | --- | --- | --- | --- | --- | --- | --- | --- | --- | --- | --- | --- | --- | --- | --- | --- | --- | --- | --- | --- | --- | --- | --- | --- | --- | --- | --- | --- | --- | --- | --- | --- | --- | --- | --- | --- | --- | --- | --- | --- | --- | --- | --- | --- | --- | --- | --- | --- | --- | --- | --- | --- | --- | --- | --- | --- | --- | --- | --- | --- | --- | --- | --- | --- | --- | --- | --- | --- | --- | --- | --- | --- | --- | --- | --- | --- | --- | --- | --- | --- | --- | --- | --- | --- | --- | --- | --- | --- | --- | --- | --- | --- | --- | --- | --- | --- | --- | --- | --- | --- | --- | --- | --- | --- | --- | --- | --- | --- | --- | --- | --- | --- | --- | --- | --- | --- | --- | --- | --- | --- | --- | --- | --- | --- | --- | --- | --- | --- | --- | --- | --- | --- | --- | --- | --- | --- | --- | --- | --- | --- | --- | --- | --- | --- | --- | --- | --- | --- | --- | --- | --- | --- | --- | --- | --- | --- | --- | --- | --- | --- | --- | --- | --- | --- | --- | --- | --- | --- | --- | --- | --- | --- | --- | --- | --- | --- | --- | --- | --- | --- | --- | --- | --- | --- | --- | --- | --- | --- | --- | --- | --- | --- | --- | --- | --- | --- | --- | --- | --- | --- | --- | --- | --- | --- | --- | --- | --- | --- | --- | --- | --- | --- | --- | --- | --- | --- | --- | --- | --- | --- | --- | --- | --- | --- | --- | --- | --- | --- | --- | --- | --- | --- | --- | --- | --- | --- | --- | --- | --- | --- | --- | --- | --- | --- | --- | --- | --- | --- | --- | --- | --- | --- | --- | --- | --- | --- | --- | --- | --- | --- | --- | --- | --- | --- | --- | --- | --- | --- | --- | --- | --- | --- | --- | --- | --- | --- | --- | --- | --- | --- | --- | --- | --- | --- | --- | --- | --- | --- | --- | --- | --- | --- | --- | --- | --- | --- | --- | --- | --- | --- | --- | --- | --- | --- | --- | --- | --- | --- | --- | --- | --- | --- | --- | --- | --- | --- | --- | --- | --- | --- | --- | --- | --- | --- | --- | --- | --- | --- | --- | --- | --- | --- | --- | --- | --- | --- | --- | --- | --- | --- | --- | --- | --- | --- | --- | --- | --- | --- | --- | --- | --- | --- | --- | --- | --- | --- | --- | --- | --- | --- | --- | --- | --- | --- | --- | --- | --- | --- | --- | --- | --- | --- | --- | --- | --- | --- | --- | --- | --- | --- | --- | --- | --- | --- | --- | --- | --- | --- | --- | --- | --- | --- | --- | --- | --- | --- | --- | --- | --- | --- | --- | --- | --- | --- | --- | --- | --- | --- | --- | --- | --- | --- | --- | --- | --- | --- | --- | --- | --- | --- | --- | --- | --- | --- | --- | --- | --- | --- | --- | --- | --- | --- | --- | --- | --- | --- | --- | --- | --- | --- | --- | --- | --- | --- | --- | --- | --- | --- | --- | --- | --- | --- | --- | --- | --- | --- | --- | --- | --- | --- | --- | --- | --- | --- | --- | --- | --- | --- | --- | --- | --- | --- | --- | --- | --- | --- | --- | --- | --- | --- | --- | --- | --- | --- | --- | --- | --- | --- | --- | --- | --- | --- | --- | --- | --- | --- | --- | --- | --- | --- | --- | --- | --- | --- | --- | --- | --- | --- | --- | --- | --- | --- | --- | --- | --- | --- | --- | --- | --- | --- | --- | --- | --- | --- | --- | --- | --- | --- | --- | --- | --- | --- | --- | --- | --- | --- | --- | --- | --- | --- | --- | --- | --- | --- | --- | --- | --- | --- | --- | --- | --- | --- | --- | --- | --- | --- | --- | --- | --- | --- | --- | --- | --- | --- | --- | --- | --- | --- | --- | --- | --- | --- | --- | --- | --- | --- | --- | --- | --- | --- | --- | --- | --- | --- | --- | --- | --- | --- | --- | --- | --- | --- | --- | --- | --- | --- | --- | --- | --- | --- | --- | --- | --- | --- | --- | --- | --- | --- | --- | --- | --- | --- | --- | --- | --- | --- | --- | --- | --- | --- | --- | --- | --- | --- | --- | --- | --- | --- | --- | --- | --- | --- | --- | --- | --- | --- | --- | --- | --- | --- | --- | --- | --- | --- | --- | --- | --- | --- | --- | --- | --- | --- | --- | --- | --- | --- |

**Figure S1: Dinucleotide profiles in 400-nt upstream regions.** Window size is 60 nts. Y-axis denotes the average occurrences of the specified dinucleotide in the 60-nt window. Plots of dinucleotides AA, AC, ..., TG, TT are arranged from top left to bottom right.


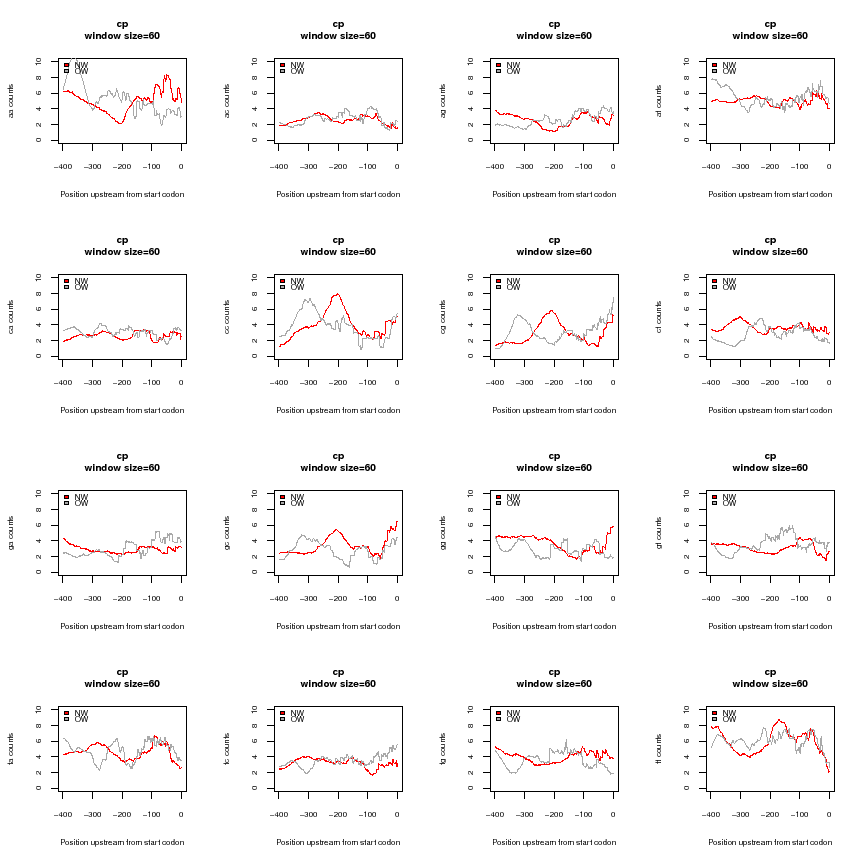


Figure S1A: Coat protein CP


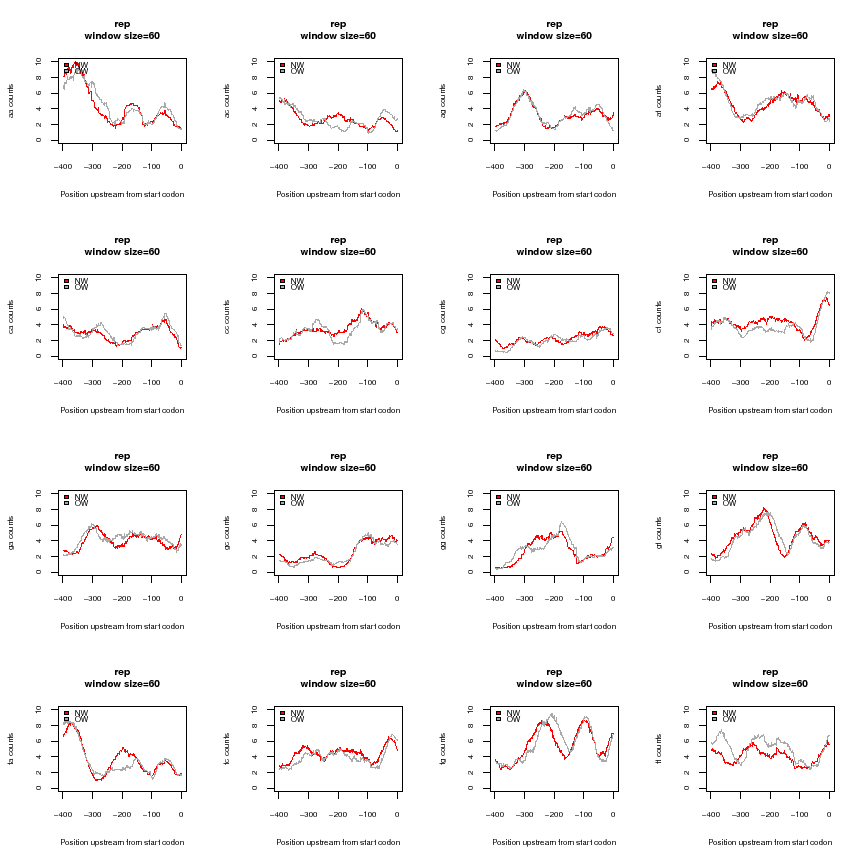


Figure S1B: Replication-associated protein REP


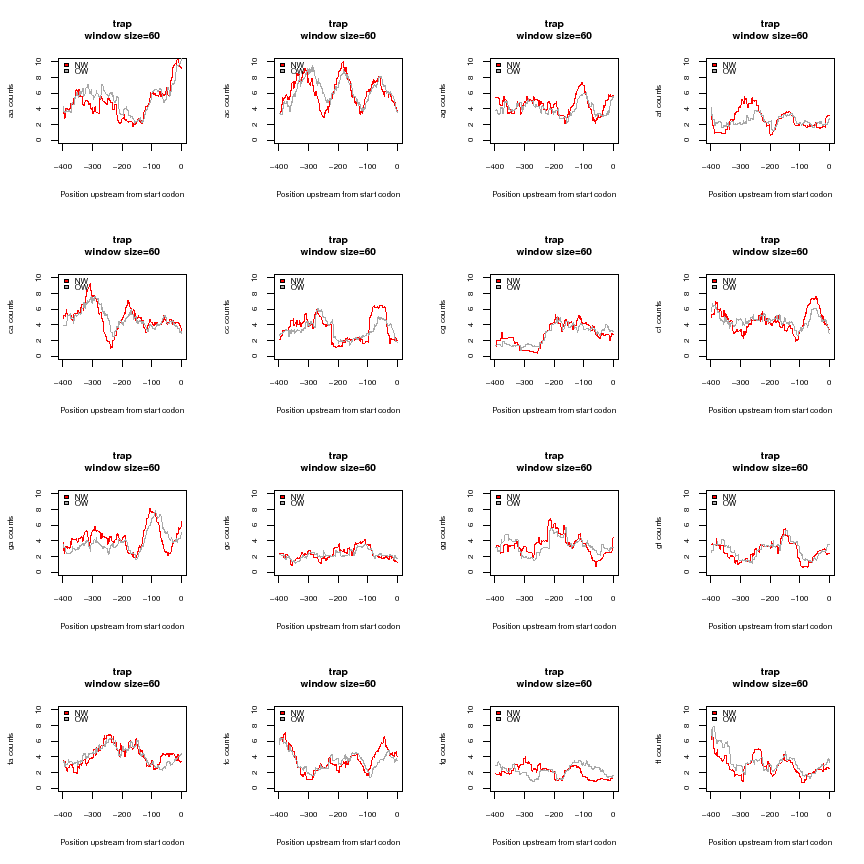


Figure S1C: Transcription activator TrAP


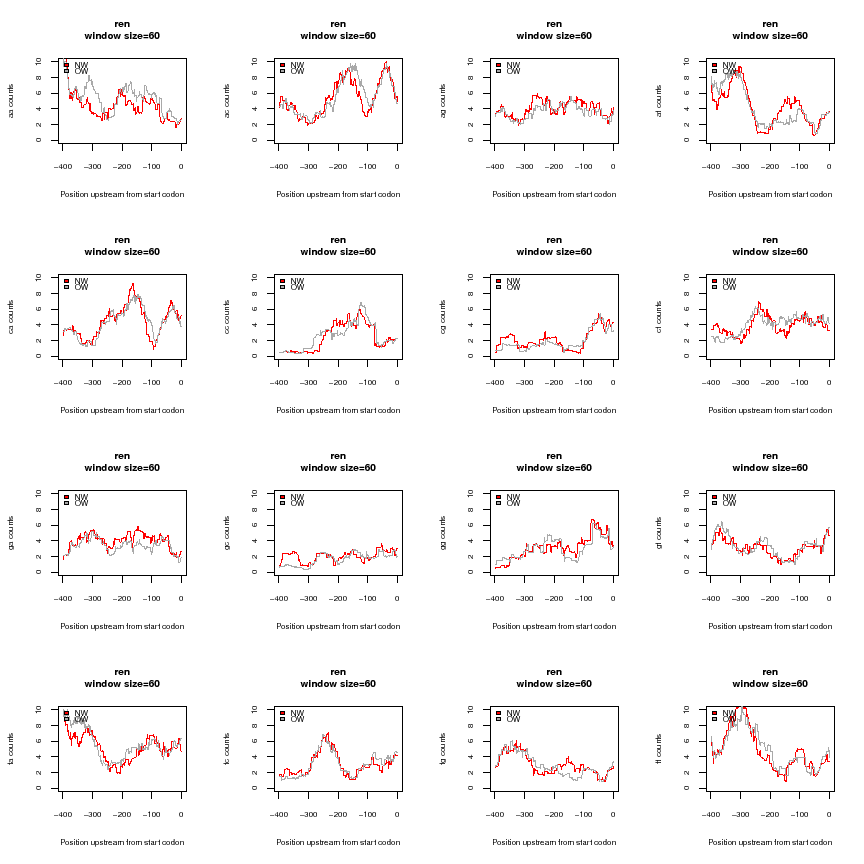


Figure S1D: Replication enhancer protein REN


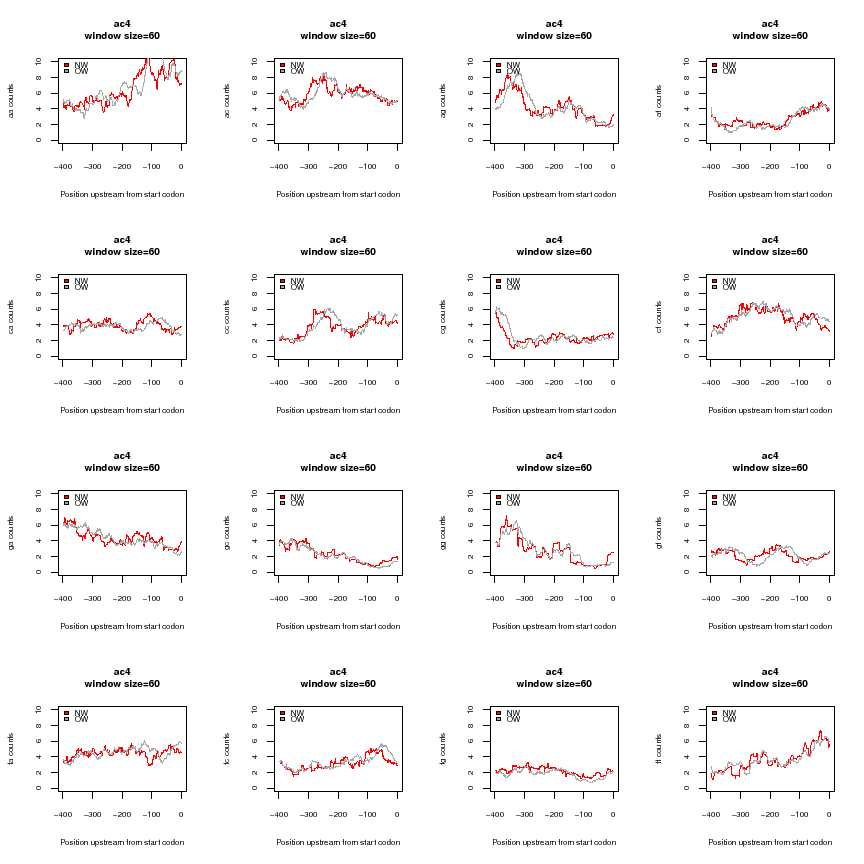


Figure S1E: AC4 protein


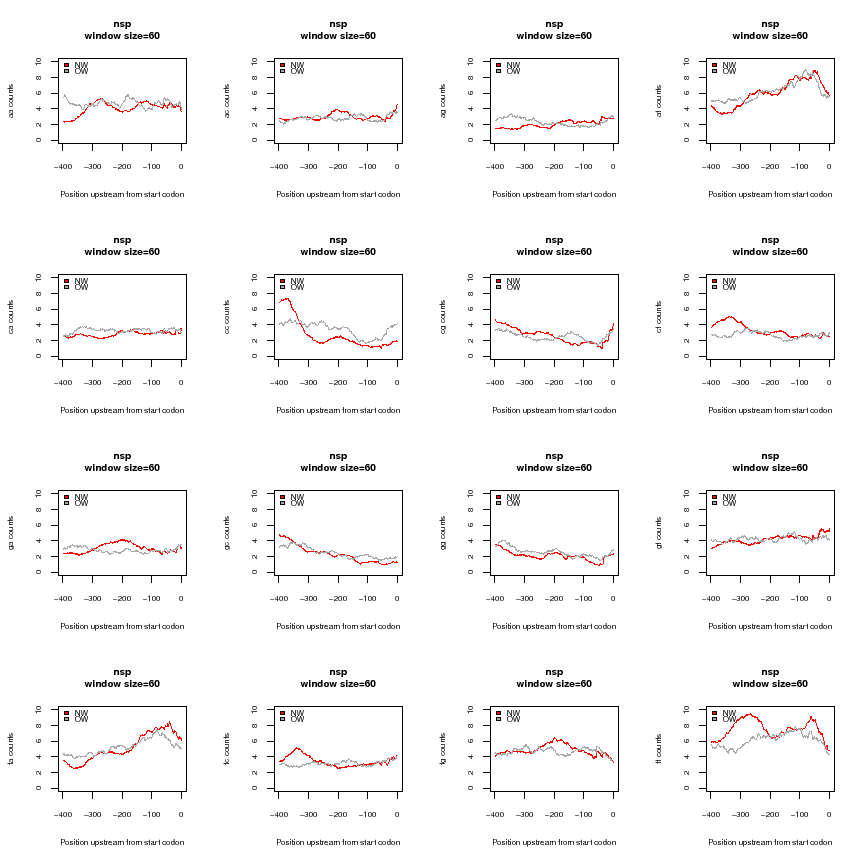


Figure S1F: Nuclear shuttle protein NSP


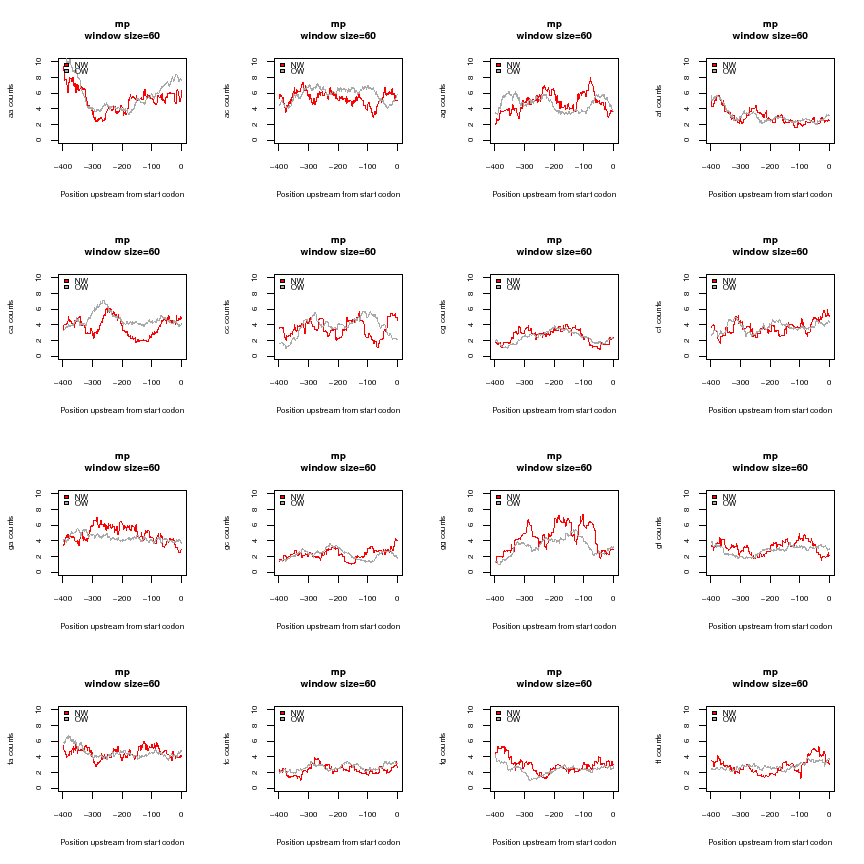


Figure S1G: Movement protein MP

**Figure S2: Cumulative dN/dS by gene**
